# Thermomechanical Noise Characterization in Fully Monolithic CMOS-MEMS Resonators

**DOI:** 10.3390/s18093124

**Published:** 2018-09-16

**Authors:** Rafel Perelló-Roig, Jaume Verd, Sebastià Bota, Jaume Segura

**Affiliations:** System Electronic Group (Physics Department), Universitat de les Illes Balears, 07122 Palma (Balearic Islands), Spain; jaume.verd@uib.es (J.V.); sebastia.bota@uib.es (S.B.); jaume.segura@uib.es (J.S.)

**Keywords:** MEMS resonators, thermomechanical noise, sensors, CMOS-MEMS

## Abstract

We analyzed experimentally the noise characteristics of fully integrated CMOS-MEMS resonators to determine the overall thermomechanical noise and its impact on the limit of detection at the system level. Measurements from four MEMS resonator geometries designed for ultrasensitive detection operating between 2-MHz and 8-MHz monolithically integrated with a low-noise CMOS capacitive readout circuit were analyzed and used to determine the resolution achieved in terms of displacement and capacitance variation. The CMOS-MEMS system provides unprecedented detection resolution of 11 yF·Hz^−1/2^ equivalent to a minimum detectable displacement (MDD) of 13 **f**m·Hz^−1/2^, enabling noise characterization that is experimentally demonstrated by thermomechanical noise detection and compared to theoretical model values.

## 1. Introduction

**Figure 1:**
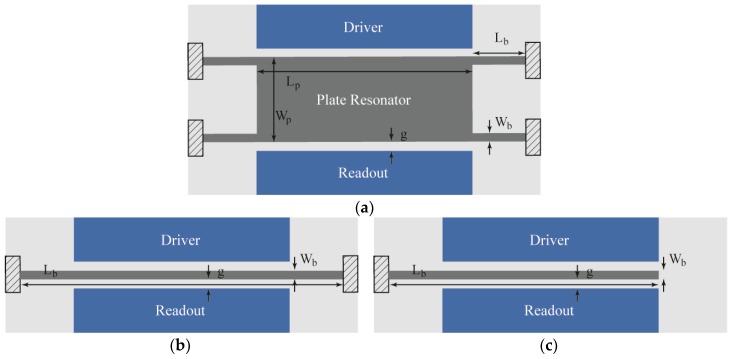
Schematics showing the three geometries analyzed in this work: (**a**) Plate-shaped resonators with two different sizes denoted as Plate-B1 and Plate-B2; (**b**) CC-Beam resonator; and (**c**) Cantilever resonator. The figure also shows, colored in dark blue, the driver-readout scheme composed by two electrodes for electrostatic actuation and capacitive readout.

**Figure 2:**
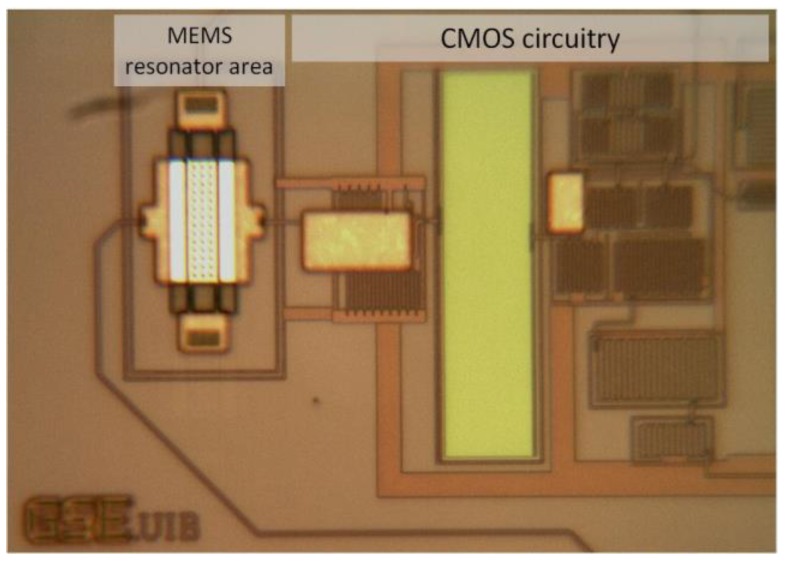
Optical image of the MEMS resonator integrated together with the CMOS amplifier using a monolithic solution. Both the resonator and CMOS circuitry are fabricated in a 0.35-μm commercial technology.

Micro and nanoelectromechanical resonators have been extensively proposed and experimentally tested for sensing purposes in the biological and chemical domains [1,2], among many others, given their extremely large mass sensitivity [3,4]. However, these systems are limited by their intrinsic noise [5], mainly thermomechanical ($V_{n,res}$), that determines the ultimate limit of detection. Additional noise may be induced when coupling the resonant structure to a readout circuit ($V_{n,amp}$), thus increasing the overall sensor noise and eventually masking the impact of thermomechanical vibrations, degrading the ultimate sensor resolution [6]. In this work, we analyze and characterize the noise contributions in four resonator geometries: two double-anchored plates (referred to as Plate-B1 and Plate-B2 in Figure 1a), a CC-Beam structure (Figure 1b) and a Cantilever (Figure 1c). All these resonators were monolithically integrated with a full-custom capacitive readout amplifier (Figure 2), achieving a $V_{n,amp}<25\;\mathrm{nV}\cdot {\mathrm{Hz}}^{-1/2}$ input referred noise (@6 MHz), using a CMOS-MEMS solution that allows a direct on-chip resonator response measurement. This signal is further processed with an ultra-low-noise high-gain amplifier so that it can also operate as a self-sustained oscillator for a variety of applications [3]. Such a low-noise amplifier scheme allows detecting the resonators thermomechanical motion, thus allowing the calibration of both the displacement sensitivity ($D_{s}$) and the minimum detectable displacement ($S_{MDD}^{1/2}$). The experimental measurements together with the theoretical model predictions show a $S_{MDD}^{1/2}=13\;fm\cdot Hz^{-1/2}$ for the Plate-B1 structure corresponding to an equivalent capacitance variation as low as $S_{\Delta C}^{1/2}=11\;\mathrm{yF}\cdot {\mathrm{Hz}}^{-1/2}$ at atmospheric pressure conditions. These results are similar to the best solutions achieved for the transduction of displacement in the micro and even nanomechanical world, that is constantly under progress [7]. Both, the $S_{MDD}^{1/2}$ and $S_{\Delta C}^{1/2}$, are more than four orders of magnitude below state-of-the-art capacitive alternatives [8,9,10,11] and piezoresistive ones [12,13], being similar to optical and microwave cavity systems [14,15,16,17]. In addition, measurements in vacuum conditions show a performance increase, achieving a $S_{MDD}^{1/2}=4.8\;\mathrm{fm}\cdot {\mathrm{Hz}}^{-1/2}$ for CC-Beam resonator, and a minimum detectable change in capacitance $S_{\Delta C}^{1/2}=2.0\;\mathrm{yF}\cdot {\mathrm{Hz}}^{-1/2}$, becoming closer to the state-of-the-art optical solutions [14,15,16,17].

## 2. Fabrication and Experimental Setup

### 2.1. Fabrication

**Figure 3:**
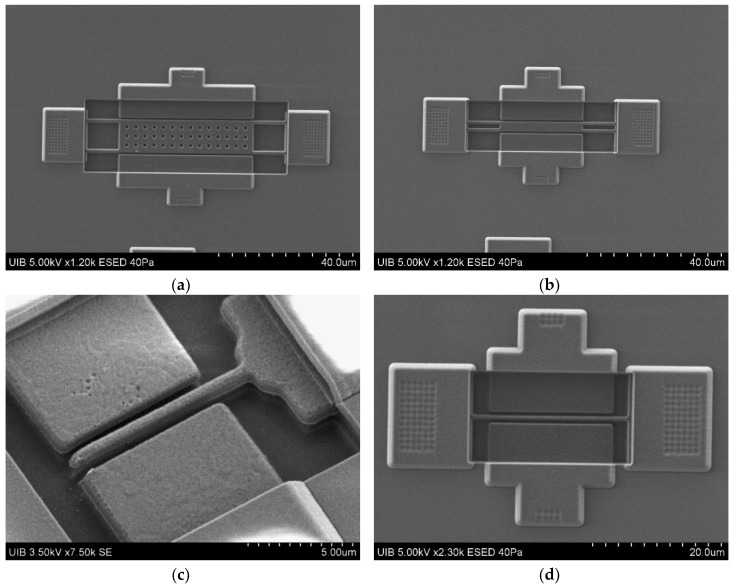
Scanning electron microscopy (SEM) images of the resonators with the driver and readout electrodes fabricated using the top metal layer of the 0.35-μm technology. (**a**) Plate-B1 resonator with a 41 μm × 10 μm platform, (**b**) Plate-B2 resonator with a 25 μm × 3 μm platform, (**c**) 10 μm long Cantilever, and (**d**) and 25 μm long CC-beam.

The MEMS resonators considered in this work were fabricated using the top metal layer (Aluminum) of a commercial 0.35-μm CMOS technology followed with a mask-less wet-etching post-CMOS step in our laboratory to release the mechanical moving parts (Figure 3) [18]. The density of the CMOS metal layer used in this work to compute the following parameters is $3000\;\mathrm{kg}/m^{3}$; and the Young’s modulus is 131 GPa. The resonator-driver gap (s) was designed to be the minimum allowed by the technology (0.6 μm) to increase the electromechanical coupling factor (η) whose formula is given in Equation (1).

(1) $$\eta =V_{MEMS}\frac{C_{0}}{s}$$

where $V_{MEMS}$ stands for the bias DC voltage applied to the resonator and $C_{0}$ the static capacitance existing between the resonator and the readout electrode given in Equation (2) that depends on the cross-sectional area (A) and the permittivity ($\varepsilon_{0}$).

(2) $$C_{0}=\frac{\varepsilon_{0}A}{s}$$

The structure thickness (t) was *0.85* μm, determined by the technology layer used. The remaining structure dimensions constitute the design parameters referred to as (see Figure 1 for reference): beam length (*L_b_*), beam width (*W_b_*), platform length (*L_p_*), and platform width (*W_p_*) taking the values given in Table 1 for the various resonators. The CC-Beam and Cantilever design parameters are reported in previous works [19,20]. The Plate Resonators are optimized to increase the effective area in order to operate as a distributed mass sensor while considering the trade-off between mass sensitivity and the maximum motional resistance of the resonator that can be sensed by the integrated amplifier [21]. From these values, we compute the required resonator electrical and mechanical parameters (see Table 2) used in further calculations along this discussion, including the theoretical model the calibrations performed from experimental results. As typically done, the resonator is modeled through a spring-mass damped system having effective mass ($M_{eff}$), spring constant (k), and a quality factor (Q) to represent the system damping. The $M_{eff}$ expression varies for each resonator geometry and resonant mode shape. For the first in-plane mode, Equations (3)–(5) give the expressions used for each structure [5].

(3) $$M_{eff}(B1,B2)=\frac{12}{2.365^{4}}\rho tL_{b}W_{b}+\rho tL_{p}W_{p}$$

(4) $$M_{eff}(\mathrm{Cantilever})=\frac{8}{1.875^{4}}\rho tL_{b}W_{b}$$

(5) $$M_{eff}(\mathrm{CC-Beam})=\frac{384}{4.730^{4}}\rho tL_{b}W_{b}$$

where ρ stands for the metal layer used to fabricate the resonators mass density. The resonators mechanical stiffness are given in Equations (6)–(8) [6].

(6) $$k(B1,B2)=\frac{EtW_{b}^{3}}{L_{b}^{3}}$$

(7) $$k(\mathrm{Cantilever})=\frac{2EtW_{b}^{3}}{3L_{b}^{3}}$$

(8) $$k(\mathrm{CC-Beam})=\frac{32EtW_{b}^{3}}{L_{b}^{3}}$$

where E refers to the Young’s modulus of the resonator material. Equations (6)–(8) for the linear spring constant don’t account for the resonator electrostatic biasing. Such dependency for which the biasing voltage contributes to decrease its value by the known spring-softening effect is given in Equations (9)–(10).

(9) $$k_{el}=\frac{V_{MEMS}^{2}}{s^{2}}C_{0}$$

(10) $$k_{eff}=k-\left|k_{el}\right|$$

Once the resonators mechanical parameters are given, we provide the electrical ones, modeling the resonator as an RLC lumped equivalent circuit [22] with a parallel parasitic capacitance ($C_{p}$) due to readout scheme. The equivalent motional resistance ($R_{M}$) is the most important parameter with an expression given in Equation (11) that depends on the electromechanical coupling defined in Equation (1).

(11) $$R_{M}=\frac{\sqrt{kM_{eff}}}{Q\eta^{2}}$$

### 2.2. Experimental Setup

**Figure 4:**
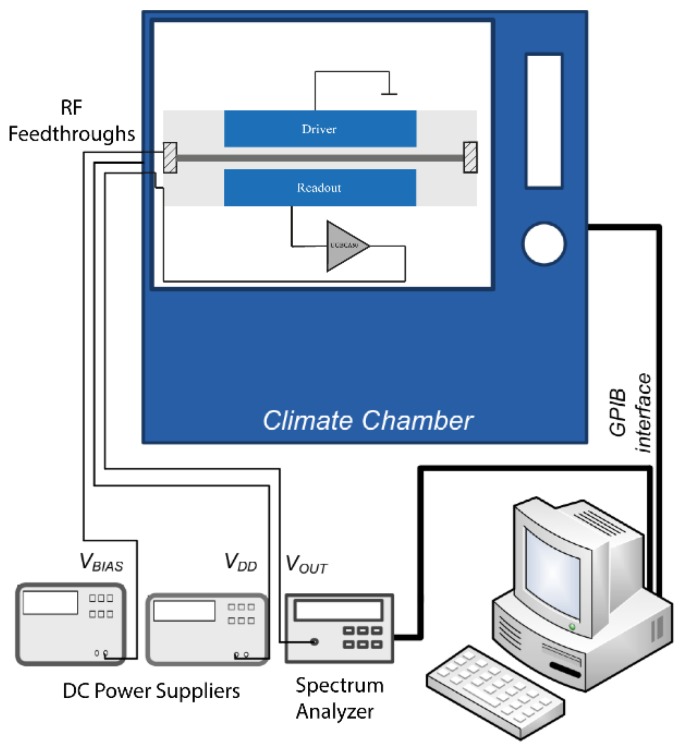
Schematic view of the experimental setup used to perform electrical measurements of CMOS-MEMS devices under controlled ambient. The climate chamber and the standalone instruments are remotely controlled.

The main goal of this work is to characterize the thermomechanical vibrations spectrum of the resonators with no driving force. For this purpose, the driving electrode was grounded and the output monitored using a spectrum analyzer while the data was recorded using a LabView^®^ program (National Instruments, Austin, TX, USA) (see Figure 4). The noise measurements were obtained using a function of the spectrum analyzer (E4407B, Agilent Technologies, Santa Clara, CA, USA), *noise marker*, specially designed for white noise like signals acquisition. The fabricated devices were tested under controlled temperature and relative humidity conditions in a climate chamber (KPK200, Feutron, Langenwetzendorf, Germany), because of its high sensitivity to both temperature and humidity [20]. Additionally, vacuum measurements were performed within an in-house made vacuum chamber keeping the temperature at a constant value.

## 3. Noise Sources Model

**Figure 5:**
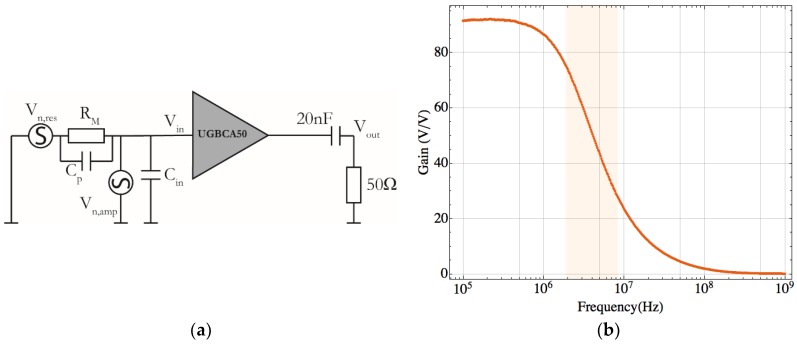
(**a**) ***E***quivalent circuit for noise analysis of the CMOS capacitive readout circuit. $V_{n,res}={\left(4k_{B}TR_{M}\right)}^{1/2}$ represents the resonator thermomechanical noise voltage per unit of bandwidth root square, and $V_{n,amp}$ is the corresponding input-referred voltage noise of the CMOS circuit. (**b**) Plot of the simulated voltage gain for the UGBCA50 amplifier. The operation region, where the gain decreases with the frequency, is highlighted in light orange.

Two main noise sources contributing to the readout signal are the resonator intrinsic thermomechanical noise ($V_{n,res}$), described by its equivalent motional resistance ($R_{M}$) [22], and the CMOS amplifier noise ($V_{n,amp}$). Figure 5a schematizes the equivalent circuit used to analyze the microelectromechanical system noise. The overall noise at the amplifier output is given by the quadrature addition of both sources multiplied by the amplifier gain (*G*). Considering the parasitic input impedance ($Z_{CI}$) of the CMOS amplifier and the impedance ($Z_{R}$) (the parallel of the motional resistance $R_{M}$ and the parasitic capacitance $C_{p}$) from the readout scheme, the resonator thermomechanical noise at the system output is given by:

(12) $$V_{out}=G\sqrt{{\left(\frac{Z_{CI}}{Z_{CI}+Z_{R}}\right)}^{2}V_{n,res}^{2}+V_{n,amp}^{2}}.$$

The thermomechanical noise spectrum is shaped by the second order frequency response of the resonator [23] according to Equation (13).

(13) $$V_{n,res}=\sqrt{4k_{B}TR_{M}}\sqrt{\frac{{\left(\frac{\omega \omega_{0}}{Q}\right)}^{2}}{{\left(\frac{\omega \omega_{0}}{Q}\right)}^{2}+{\left(\omega^{2}-\omega_{0}^{2}\right)}^{2}}}$$

where $k_{B}$ is the Boltzmann constant, *T* is the absolute temperature, $\omega_{0}$ is the angular resonance frequency, and Q the quality factor. Equations (12) and (13) indicate that the thermomechanical noise peak at the circuit output increases as long as $R_{M}$ decreases. The thermomechanical noise at resonance can be experimentally measured, assuming a unity signal-to-noise ratio as the detection threshold level, only if the amplifier noise contribution at this frequency is below the thermomechanical noise according to:

(14) $$V_{n,amp}<\sqrt{4k_{B}TR_{M}}\frac{Z_{CI}}{Z_{CI}+Z_{R}}.$$

Notice that Equation (14) only depends on $R_{M}$ and *T* as far as $Z_{CI}$ and $C_{p}$ are set by the readout driver layout and the CMOS amplifier input parasitic capacitance. For a determined operation temperature, there is a range of $R_{M}$ values that make the amplifier noise to be smaller than the thermomechanical noise peak. The values obtained at 20 °C are given in Table 3 indicating that the thermomechanical noise detection is possible for all the structures using bias voltages below 50 V.

## 4. Results

**Figure 6:**
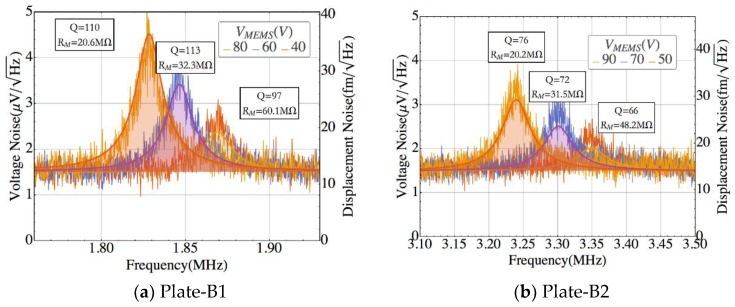

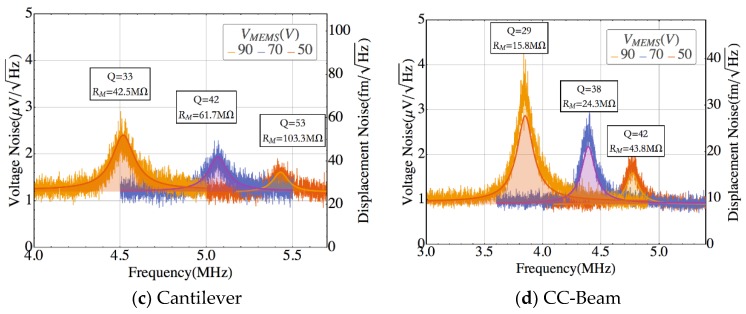
Measured voltage noise spectrum operated at ambient pressure and 20% relative humidity, compared to the theoretical model (solid-filled line) for various bias voltages at 20 °C. The values given for Q and $R_{M}$ are derived from experimental data fitting. (**a**) Plate-B1, (**b**) Plate-B2, (**c**) Cantilever, and (**d**) CC-Beam.

**Figure 7:**
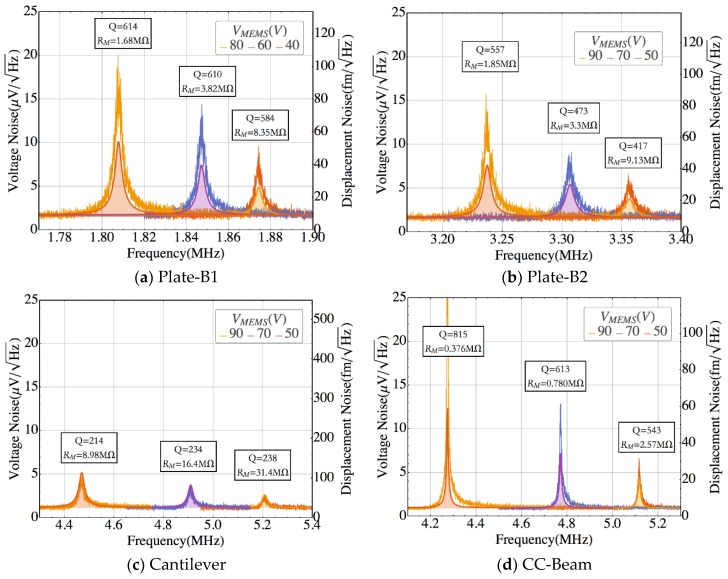
Measured voltage noise spectrum operated at vacuum conditions (P = 10^−4^ mbar), compared to the theoretical model (solid-filled line) for various bias voltages at 20 °C. The values given for Q and $R_{M}$ are derived from experimental data fitting. (**a**) Plate-B1, (**b**) Plate-B2, (**c**) Cantilever, and (**d**) CC-Beam.

The model predictions were experimentally confirmed for all the geometries thus corroborating the noise capabilities of these CMOS-MEMS devices. The thermomechanical noise was observed at 20 °C and bias voltages ranging from 40 V to 90 V both under ambient pressure (Figure 6) and vacuum conditions (Figure 7). Furthermore, we also verified experimentally that the resonance peak shape obtained without driving any electrical force fits the theoretical model accurately enough to confirm the noise source being caused by thermomechanical fluctuations. The experimental data depicted in Figure 6 and Figure 7 is labeled with the Q and $R_{M}$ obtained by fitting the data points to a Lorentzian curve. Additionally, we also show the predictions from the theoretical model (solid-line) emphasizing the good matching. The predicted behavior was experimentally corroborated for all the geometries proposed, each one having a different quality factor and resonant peak value (due to the dependence of these parameters with $R_{M}$) highlighting the accuracy of the model developed and the nature of thermomechanical vibrations of the signal measured. The boundary values for $R_{M}$ are given in Table 3, as well as the theoretical value of $R_{M}$ and its experimentally fitted one. The measured output voltage noise depends also on the CMOS amplifier gain that decreases with the frequency (Figure 5b).

The experimental data shows that the frequency decreases as the bias voltage increases due to the well-known spring softening effect. Plate-B1 resonator exhibits the highest peak value (Figure 6) in agreement with its small resonance frequency—the amplifier gain is inversely proportional to the operation frequency (Figure 5b)—and also its small $R_{M}$ (see Table 3). This behavior is corroborated for the remaining geometries: the Cantilever presents the smallest resonance peak, being the structure with the largest $R_{M}$ and frequency, followed by the CC-Beam and the Plate-B2 structure. As a general rule, the higher the $R_{M}$ value the larger the operating frequency, resulting in a smaller thermomechanical noise level at resonance, except for the CC-beam, where a trade-off between frequency of operation and $R_{M}$ is presented.

The same behavior was found when operating the resonators under vacuum conditions (Figure 7). In this case, the measured resonant peaks were larger for all geometries due to the increased Q-factor value (the air-damping losses are significantly reduced [24]). 

### Ultimate Resolution Limit

The system displacement sensitivity ($D_{s}$), defined as the system output voltage for unitary equivalent displacement, is calibrated using the resonator thermomechanical noise spectrum. In summary, we compute the displacement sensitivity as the ratio between the measured output voltage noise at resonance and the theoretical displacement noise ($S_{x}^{1/2}$). Then, this result is used to compute the minimum detectable displacement ($S_{MDD}^{1/2}$) from the spectrum bottom noise. Finally, the capacitance sensitivity ($S_{\Delta C}^{1/2}$) is derived from the $S_{MDD}^{1/2}$ and the change of capacitance with resonator displacement (∂C(x)/∂x). The theoretical displacement noise at resonance is given by [25] and represents the effective amplitude of the undriven resonator vibrations caused only by thermal noise; it has units of m·Hz^−1/2^.

(15) $$S_{x}^{1/2}=\sqrt{\frac{4k_{b}TQ}{\omega_{0}^{3}M_{eff}}}$$

From voltage noise experimental measurements, we computed the contribution of the displacement noise to the output signal—output amplifier signals are denoted with the superscript *out*—as follows: the overall noise measured at the amplifier output ($V_{n}^{out}$), given in Equation (12), has a contribution coming from the amplifier noise ($V_{n,amp}^{out}$) and also from the resonator vibrations ($V_{n,res}^{out}$). We subtracted $V_{n,amp}^{out}$ from $V_{n}^{out}$ to obtain $V_{n,res}^{out}$ with units of V·Hz^−1/2^ being directly related to $S_{x}^{1/2}$.

(16) $$V_{n,res}^{out}=\sqrt{{\left(V_{n}^{out}\right)}^{2}-{\left(V_{n,amp}^{out}\right)}^{2}}$$

Next, we took the computed $V_{n,res}^{out}$ from experimental data and the value obtained for $S_{x}^{1/2}$ to calculate the displacement sensitivity ($D_{s}$) as given in Equation (17)—with units of $Vm^{-1}$—representing the transduction from displacement to output voltage of the whole system.

(17) $$D_{s}=\frac{V_{n,res}^{out}}{S_{x}^{1/2}}$$

After obtaining the transduction factor as a displacement sensitivity, we define the minimum detectable displacement ($S_{MDD}^{1/2}$) getting the system bottom flat noise from the output voltage spectrum, equal to $V_{n,amp}^{out}$ divided by the $D_{s}$ value obtained from Equation (17). In this sense, the $S_{MDD}^{1/2}$ represents the equivalent resonator vibration amplitude providing an output voltage equal to the noise given by the CMOS amplifier, its units are m·Hz^−1/2^.

(18) $$S_{MDD}^{1/2}=\frac{V_{n,amp}}{D_{s}}$$

Since we have computed the $S_{MDD}^{1/2}$ and this device makes use of a capacitive readout scheme to convert the displacement into an electrical signal, we obtain the minimum detectable capacitance change ($S_{\Delta C}^{1/2}$). We first obtain a relationship between the resonator displacement (x) and the change in capacitance (∂C/∂x) used to compute the $S_{\Delta C}^{1/2}$ considering the electrical model to be a parallel-plate capacitance.

(19) $$C\left(x\right)=\frac{\varepsilon_{0}A}{s-x}$$

We take the expression for the parallel-plate capacitance and obtain the partial derivative around the equilibrium position; assuming that x≪s we approximate the capacitance sensitivity to displacement as follows:

(20) $$\frac{\partial C\left(x\right)}{\partial x}=\frac{-\varepsilon_{0}A}{{\left(s-x\right)}^{2}}\approx -\frac{C_{0}}{s}.$$

Finally, we use Equation (20) and the $S_{MDD}^{1/2}$ to compute $S_{\Delta C}^{1/2}$ which has units of $F\cdot {\mathrm{Hz}}^{-1/2}$. It is important to highlight that this parameter represents the minimum capacitance variation that our system is capable to measure.

(21) $$S_{\Delta C}^{1/2}=\frac{C_{0}}{s}S_{MDD}^{1/2}$$

The double-anchored plate resonator B1 gave $V_{n,res}^{out}=4.2\;\mu V\cdot {\mathrm{Hz}}^{-1/2}$, corresponding to an estimated noise displacement of $S_{x}^{1/2}=34\;\mathrm{fm}\cdot {\mathrm{Hz}}^{-1/2}$ resulting in an achieved displacement sensitivity of $D_{s}=123\;\mathrm{MV}\cdot m^{-1}$. This result can be validated by comparison to the theoretical value obtained from the capacitance change and motional current ($I_{M}$).

(22) $$V_{out}=G_{T}I_{M}=G_{T}V_{DC}\frac{\partial C}{\partial x}\frac{\partial x}{\partial t}=G_{T}V_{DC}\frac{C_{0}}{s}\omega \langle x\rangle =G_{T}\eta \omega \langle x\rangle$$

(23) $$D_{S}\left(theo\right)=\frac{\partial V}{\partial x}=G_{T}\eta \omega$$

where $G_{T}$ stands for the transimpedance gain in units of Ω and x is the vibration amplitude. The theoretical value for the displacement sensitivity from Equation (23) using a bias voltage of 80 V and $G_{T}=130\;M\Omega$, given the same conditions for B1 than the experimental value gives $D_{s}\left(theo\right)=104\;\mathrm{MV}\cdot m^{-1}$, matching accurately enough the result derived from experimental measurements ($D_{s}=123\;\mathrm{MV}\cdot m^{-1})$. The large value shown for the displacement sensitivity is mainly achieved thanks to a high-gain amplifier, a large electromechanical coupling resonator-electrode and to an operating frequency in the range of MHz.

Therefore, the results achieved for $S_{MDD}^{1/2}$ and $S_{\Delta C}^{1/2}$ values in the case of the plate resonator B1 operated at atmospheric pressure are $S_{MDD}^{1/2}=13\;\mathrm{fm}\cdot {\mathrm{Hz}}^{-1/2}$ and $S_{\Delta C}^{1/2}=11\;\mathrm{yF}\cdot {\mathrm{Hz}}^{-1/2}$, being further improved when the devices operated under vacuum conditions; mainly due to the quality factor Q improvement. The displacement sensitivity for the plate resonator B1 in vacuum was almost doubled to $D_{s}=190\;\mathrm{MV}\cdot m^{-1}$, thus decreasing the minimum detectable displacement to $S_{MDD}^{1/2}=8.5\;\mathrm{fm}\cdot {\mathrm{Hz}}^{-1/2}$, and the equivalent capacitance change to $S_{\Delta C}^{1/2}=7.3\;\mathrm{yF}\cdot {\mathrm{Hz}}^{-1/2}$. The results for the other three structures are given in Table 4, together with a review of the state-of-the-art resonator parameters for various readout techniques. 

In the case of the monolithic solution and capacitive readout [9,11], as far as we know, this work proves to deliver the best results in terms of $S_{MDD}^{1/2}$ and $S_{\Delta C}^{1/2}$ even when comparing to non-monolithic solutions [8,10], that report a $S_{MDD}^{1/2}\sim \;\mathrm{pm}\cdot {\mathrm{Hz}}^{-1/2}$ and $S_{\Delta C}^{1/2}\sim \mathrm{zF}\cdot {\mathrm{Hz}}^{-1/2}$, which are $10^{3}–10^{4}\times$ larger than the results provided in this work. Similarly, the outcomes also improve the results obtained for piezoresistive monolithic systems in terms of $S_{MDD}^{1/2}$ [12,13] by 100x. Finally, our results also improve by 10× the outcomes achieved by opto-mechanical systems, both the optical readout and the microwave cavity solution [16,17], with the exception of the works by Ding [14] and Zhang [15] that provide a $S_{MDD}^{1/2}$ that is 10× better than our result. These two-latter works exploit the advantages of the optical readout system that allows operation at very-high frequency, with the limitation of not being easily integrated as a monolithic solution. 

Therefore, the solution proposed in this work provides ultra-high displacement and capacitance resolution by means of a CMOS-MEMS monolithic approach in the sub-micrometer range both in air and vacuum conditions thanks to the overall system integration that minimizes the non-desired parasitic contributions. Depending on the application, the vacuum improvement is close to being 2× better which does not represent a significant improvement. This is a key when developing mass sensors that typically work at atmospheric pressure conditions since the resonator surface must be easy accessible to deposit the mass to be sensed [19]. This also applies to volatile or gas sensing trough gravimetric techniques that must operate at atmospheric conditions.

## 5. Conclusions

The results reported here demonstrate that the ultra-low-noise capacitive readout system integrated monolithically with the mechanical resonator achieves to resolve the thermomechanical fluctuations of four different resonators. The minimum capacitance change detected is close to the *yF* barrier being measurable both in air and vacuum conditions. Such a capacitance variation corresponds to an equivalent resonator displacement being in the *fm* range. This is possible thanks to the monolithically integrated CMOS-MEMS solution that reduces significantly the interconnect parasitics between the mechanical sensing part and the amplifier electronics—making use of a mature low-cost commercial 0.35-μm CMOS technology—and thanks to the ultra-low-noise amplifier design. Furthermore, these outcomes are orders of magnitude better than other monolithic CMOS-MEMS solutions and close to the opto-mechanical state-of-the-art devices as shown in Table 4, providing $S_{MDD}^{1/2}$ values in the order of *fm*. We have also obtained outstanding displacement sensitivities reaching values in the order of hundreds $\mathrm{MV}\cdot m^{-1}$ thanks to the high electromechanical transduction achieved.

We have given proof that the un-driven measured output spectrum corresponds to thermomechanical noise coming from the Brownian motion of the resonators; the theoretical model developed matches the captured experimental data for all geometries accurately enough, emphasizing the frequency shift when changing the bias voltage due to the spring softening effect. Moreover, the theoretical model predicted the range of motional resistances allowed to be able to sense the resonator thermomechanical motion, which emphasizes the thermomechanical nature of the measured noise.

In summary, this work demonstrates feasible ultrasensitive resonators, enabling the development of compact and light system on-chip sensing devices exploiting the ultimate limits of the sensor resolution for different applications that must operate in atmospheric conditions. Additionally, vacuum operation even improves in the $S_{MDD}^{1/2}$ and $S_{\Delta C}^{1/2}$, demonstrating the viability of focusing on other applications related to displacement measurement that do allow operating with vacuum packaged systems. 

## Figures and Tables

**Table 1:** Geometrical parameters of the different structures reported in this work.

Structure	*L_b_*(μm)	*W_b_*(μm)	*L_p_*(μm)	*W_p_*(μm)
Plate-B1	10	0.8	41	10
Plate-B2	10	0.8	25	3.0
Cantilever	10	0.6	-	-
CC-Beam	25	0.6	-	-

**Table 2:** Resonator parameters obtained from the theoretical expressions. The spring constant value does not consider the spring-softening effect, and the motional resistance and coupling are computed at 50 V biasing voltage except for Plate-B1 is computed at 40 V biasing. The quality factor and resonance frequency are obtained from experimental data presented below, given the same biasing voltage. * Refers to vacuum (1 μTorr).

Structure	*M_eff_* (pg)	*K* (Nm^−1^)	$\eta \;\left(VFm^{-1}\right)$	*R_M_* (MΩ)	*F* (MHz)	*Q*
Plate-B1	967	57.0	4.28·10^−8^	65.3	1.86	97
				10.4 *	1.87 *	610 *
Plate-B2	199	57.0	2.61·10^−8^	68.7	3.35	66
				11.1 *	3.36 *	446 *
Cantilever	9.90	16.0	0.836·10^−8^	107.4	5.43	53
				23.9 *	5.21 *	238 *
CC-Beam	29.3	49.3	2.01·10^−8^	71.5	4.78	42
				5.53 *	5.12 *	543 *

**Table 3:** Motional resistance bounds providing thermomechanical noise values at resonance beyond the circuit noise together with the theoretical resistance and the experimentally fitted ones. The theoretical predictions are computed assuming atmospheric pressure operation. Bias voltage is 50 V for all resonators, except for Plate-B1 that is 40 V.

Structure	$\;R_{M,min}\left(M\Omega \right)$	$R_{M,max}\left(M\Omega \right)$	$R_{M,theo}\left(M\Omega \right)$	$R_{M,exp}\left(M\Omega \right)$
Plate-B1	0.053	154	65.3	60.1
Plate-B2	0.045	92.5	68.7	48.2
Cantilever	0.070	90.4	107.4	103.3
CC-Beam	0.054	61.3	71.5	43.8

**Table 4:** State-of-the-art of MEMS resonator sensors including various fabrication approaches and readout systems. The results provided by this work * are obtained using the largest bias voltage in each case. The second row of the devices related to this work refers to the results obtained in vacuum, where the Q increase improves the displacement sensitivity.

Reference	Detection/SoC	$f_{0}$	$V_{n,amp}^{out}$ (V·HZ^−1/2^)	$S_{x}^{1/2}$ (m·HZ^−1/2^)	$D_{S}\;$ (Vm^−1^)	$S_{MDD}^{1/2}\;$ (m·Hz^−1/2^)	$\;S_{\Delta C}^{1/2}$ (FHZ^−1/2^)
B1 *	Capacitive/Monolithic	1.85 MHz	1.6 × 10^−6^	3.4 × 10^−14^	1.2 × 10^8^	1.3 × 10^−14^	1.1 × 10^−23^
				8.0 × 10^−14^	1.9 × 10^8^	8.5 × 10^−15^	7.3 × 10^−24^
B2 *	Capacitive/Monolithic	3.30 MHz	1.5 × 10^−6^	2.4 × 10^−14^	1.1 × 10^8^	1.4 × 10^−14^	7.4 × 10^−24^
				6.1 × 10^−14^	1.8 × 10^8^	8.3 × 10^−15^	4.3 × 10^−24^
Cantilever *	Capacitive/Monolithic	5.00 MHz	1.2 × 10^−6^	4.7 × 10^−14^	4.6 × 10^7^	2.6 × 10^−14^	4.3 × 10^−24^
				11 × 10^−14^	4.6 × 10^7^	2.6 × 10^−14^	4.4 × 10^−24^
CC-Beam *	Capacitive/Monolithic	4.50 MHz	1.0 × 10^−6^	2.9 × 10^−14^	1.0 × 10^8^	9.7 × 10^−15^	4.0 × 10^−24^
				9.5 × 10^−14^	2.1 × 10^8^	4.8 × 10^−15^	2.0 × 10^−24^
[8]	Capacitive/NOT	21 kHz	3.0 × 10^−7^	-	-	-	2.7 × 10^−21^
[9]	Capacitive/Monolithic	1.5 MHz	3.5 × 10^−8^	-	-	1.5 × 10^−11^	2.6 × 10^−21^
[10]	Capacitive/Hybrid	13 MHz	5.0 × 10^−7^	-	-	-	1.3 × 10^−19^
[11]	Capacitive/Monolithic	5.3 kHz	2.5 × 10^−5^	-	-	-	1.6 × 10^−20^
[12]	Piezo/Monolithic	126 MHz	1.5 × 10^−9^	-	3.8 × 10^4^	3.9 × 10^−14^	-
[13]	Piezo/Hybrid	19 MHz	1.3 × 10^−8^	-	4.2 × 10^4^	3.1 × 10^−13^	-
[14]	Optical/NOT	860 MHz	1.1 × 10^−6^	-	5.6 × 10^10^	2.0 × 10^−17^	-
[15]	Optical/NOT	5.4 GHz	-	-	-	1.1 × 10^−17^	-
[16]	Optical/NOT	13 MHz	2.0 × 10^−8^	-	2.0 × 10^7^	1.0 × 10^−15^	-
[17]	MW Cavity/NOT	54 MHz	-	-	-	1.3 × 10^−15^	-
